# Multiple Chronic Conditions and Use of Complementary and Alternative Medicine Among US Adults: Results From the 2012 National Health Interview Survey

**DOI:** 10.5888/pcd13.150501

**Published:** 2016-05-05

**Authors:** Laura Falci, Zaixing Shi, Heather Greenlee

**Affiliations:** Author Affiliations: Laura Falci, Zaixing Shi, Department of Epidemiology, Mailman School of Public Health, Columbia University, New York, New York; Heather Greenlee, Department of Epidemiology, Mailman School of Public Health, Columbia University, and Herbert Irving Comprehensive Cancer Center, Columbia University Medical Center, New York, New York.

## Abstract

**Introduction:**

More than 25% of American adults report having 2 or more chronic conditions. People with chronic conditions often use complementary and alternative medicine (CAM) for self-care and disease management, despite a limited evidence base.

**Methods:**

Data from the 2012 National Health Interview Survey (NHIS) (n = 33,557) were analyzed to assess associations between presence of multiple chronic conditions (n = 13) and CAM use, using multivariable relative risk and linear regressions weighted for complex NHIS sampling. CAM use was defined as self-reported use of one or more of 16 therapies in the previous 12 months.

**Results:**

Chronic conditions were common. US adults reported one (22.3%) or 2 or more (33.8%) conditions. Many used at least one form of CAM. Multivitamins, multiminerals, or both (52.7%); vitamins (34.8%); and minerals (28.4%) were the most common. Compared with adults with no conditions, adults with 2 or more conditions were more likely to use multivitamins or multiminerals or both, vitamins, minerals, nonvitamins or herbs, mind–body therapies, chiropractic or osteopathic manipulation, massage, movement therapies, special diets, acupuncture, naturopathy, or some combination of these therapies (*P* <.003).

**Conclusion:**

People with multiple chronic conditions have a high prevalence of CAM use. Longitudinal studies are needed to understand the association between CAM use and chronic disease prevention and treatment.

## Introduction

In 2012, more than 25% of US adults reported having 2 or more chronic conditions, which increased from 22% in 2001 (1,2). Because of this increase, the Department of Health and Human Services (DHHS) formed the Multiple Chronic Conditions Working Group to compile a list of chronic conditions to improve disease management and quality of life for people with chronic comorbid conditions (3,4).

People with multiple chronic conditions face health care burdens because of the complexity of coordinating disease management, including treatment by medical professionals and self-care (3). Prior studies show that people with chronic conditions (5–14) often use complementary and alternative medicine (CAM) therapies as part of disease management. CAM therapies refer to a number of approaches not part of mainstream conventional medicine, used either in complement with or in lieu of standard medical treatments (15). Studies to date suggest that people with chronic conditions are more likely to use CAM, and people with additional conditions have an increased likelihood of overall CAM use (5,7,8,9,11,16–18). However, one study among patients with chronic liver disease showed an inverse association between additional comorbidities and current CAM use (19).

The aim of this study was to determine the association between use of CAM therapies and multiple chronic conditions in a large nationally representative population of US adults. To our knowledge, no studies have examined specific CAM therapy use with comorbid conditions. Studies examining comorbid conditions and CAM use have collapsed CAM into any use versus no use, whereas in reality, CAM therapies represent a heterogeneous group of behaviors that differ in type, usage, and bodies of evidence on efficacy. Understanding use of specific CAM modalities among people with multiple chronic conditions could increase knowledge about CAM therapies and disease management.

## Methods

The 2012 National Health Interview Survey (NHIS) (20) is a cross-sectional household survey conducted annually by the Centers for Disease Control and Prevention of the US noninstitutionalized, civilian population. The NHIS uses a complex sampling procedure to obtain a nationally representative sample (21). Since 2002 and every 5 years thereafter, the NHIS has included a survey supplement on CAM use. The 2012 NHIS data set included 34,525 adults (people aged 18 or older). People were excluded from this analysis if they had missing data on all CAM variables (n = 968), leaving a final sample size of 33,557.

The number of chronic conditions was calculated by using the list developed by the Multiple Chronic Conditions Working Group (4). Of the 20 chronic conditions listed by the working group, 13 conditions were ascertained in the NHIS 2012 interview (4,20). The chronic condition variables selected from the 2012 data set were those that best reflected the definition of a chronic condition (4). Participants self-reported having ever been told by a physician they had the following conditions: hypertension 2 times or more, cancer (excluding nonmelanoma skin cancer), chronic obstructive pulmonary disease (COPD, emphysema, or chronic bronchitis in the past 12 months), diabetes, hepatitis, coronary heart disease (CHD), stroke, arthritis, depression, high cholesterol; and any of the following conditions in the past 12 months: asthma attack, weak or failing kidneys, or substance abuse (20). In 2012, because of the CAM supplement being included in the NHIS, additional conditions were assessed that are on the DHHS list but not usually ascertained, including high cholesterol, depression, and substance abuse. A composite variable summed the number of chronic conditions each subject reported (range, 0–13). It was conservatively assumed that any missing value for a single condition would be recoded as a “no” response. The composite variable was categorized into 3 levels: none, 1, and 2 or more of the 13 selected chronic conditions.

The NHIS contains dichotomous (yes/no) information on use of 20 different CAM therapies: body-based therapies including chiropractic or osteopathic manipulation, massage, acupuncture, and movement therapy; mind–body therapies including yoga, tai chi, qi gong, energy healing therapy, hypnosis, and biofeedback; alternative therapies including Ayurvedic medicine, chelation therapy, craniosacral therapy, homeopathy, naturopathy, and traditional healing; dietary supplements including vitamins, minerals, multivitamin or multimineral, and other nonvitamin or herbal therapies; and special diets. Energy healing therapy, biofeedback, hypnosis, yoga, tai chi, qi gong, and mind–body therapies such as guided relaxation were collapsed into one mind–body therapy variable because they are similar behavioral CAM therapies. Therefore, this analysis examined 16 dichotomous CAM therapy outcome variables, defined as using a therapy or seeing a practitioner for the modality or both in the past 12 months. A CAM use index was created by summing the number of CAM therapies each individual used (range, 0–16). It was assumed that any missing value for a single therapy would be recoded as a “no” response.

The definition of CAM therapies is largely based on the classifications of the National Center for Complementary and Integrative Health (15), though these analyses also include the use of vitamins, minerals, and multivitamins because of their high prevalence of use. The inclusion of vitamins, minerals, and multivitamins in the definition of CAM varies in the literature. More than 50% of the US population uses dietary supplements including multivitamins or minerals and singular vitamin and mineral supplements, and this use has increased over the past 20 years (22). Because use is so widespread and the risk for supplements to interact with standard pharmaceutical treatments is high (23), it is important to describe all supplement use. Therefore, we analyzed these individual therapies and 3 CAM indices; all CAM, excluding multivitamins or minerals, and further excluding singular vitamin and mineral supplements.

Demographic and psychosocial characteristics were examined for confounding effects. A priori confounders included respondent-reported race/ethnicity, sex, age, employment status in the previous year (yes/no), imputed family income, and highest level of education. Hypothesized confounders included region, body mass index (BMI), marital status, and the following in the past 12 months: feeling frequently stressed and/or anxious (yes/no), perceived health status (fair or poor vs excellent to good), and fatigue (yes/no).

Frequencies analysis and bivariable and multivariable analyses were performed to assess the association between the presence of multiple chronic conditions and CAM use. Each CAM therapy was analyzed in separate unadjusted and adjusted Poisson regression models with a robust error variance that estimated the relative risk of CAM use, comparing participants with 1 and 2 or more chronic conditions with participants with no chronic conditions (the reference group). Bonferroni procedures (24) were used to account for multiple comparisons; the standard α level of 5% was divided by 17 (the total number of specific CAM therapies plus the CAM index) to create a corrected α level of .003. Data on chelation therapy was not shown because of the small sample size (n = 17). The relationship between the CAM index and multiple chronic conditions was determined by using a linear regression model adjusted for confounders. Confounders to be included in the final models were identified by using the minimally adjusted models, which included all a priori confounders. Hypothesized confounders were added to minimally adjusted models for all of the separate CAM outcomes. The variables that appreciably changed the parameter estimates by 10% were included in all of the final models. Multicollinearity of predictors was assessed for the final adjusted models by examining tolerance and variance inflation factor characteristics in a linear regression model (25). Tolerance and variance inflation factors are statistical values that describe the percentage by which 1 predictor is explained by the other predictors in the model. Values of tolerance below 10% and variance inflation factor above 10 indicate potential collinearity. Missing covariates were not imputed and were excluded from individual regression models. All regression analyses were weighted on the basis of the complex NHIS sampling survey procedure (26), using SAS software version 9.3 (SAS Institute Inc).

## Results

Chronic conditions were common in the US population as sampled in the 2012 NHIS, where 22.3% of adults reported 1 condition and 33.8% reported 2 or more conditions; therefore, more than half (56.1%) reported at least 1 chronic condition (Table 1). Of the participants with 2 or more chronic conditions, most had 2 conditions (42.3%), followed by 3 (27.5%) and 4 (15.9%) conditions (data not shown). The average age of participants was 48 years and most adults were non-Hispanic white (67.2%), female (51.8%), and employed (66.5%). Compared with adults with no chronic conditions, adults with multiple chronic conditions were older, had a lower income, were less educated, were unemployed, were more likely to be obese, and reported having worse perceived health status and being frequently stressed or anxious.

**Table 1:** Population Characteristics by Number of Chronic Conditions, National Health Interview Survey, 2012

Characteristic	No. of Chronic Conditions								*P* b
	Total Study Population		0 Chronic Conditions		1 Chronic Condition		≥2 Chronic Conditions		
	n	% (95% CI)^a^	n	% (95% CI)^a^	n	% (95% CI)^a^	n	% (95% CI)^a^	
Totals	33,557	100 (NA)	13,790	43.9 (43.1–44.6)	7,427	22.3 (21.7–22.9)	12,340	33.8 (33.1–34.5)	NA
**Race/ethnicity**									
Hispanic	5,738	15.0 (14.3–15.7)	3,155	20.0 (18.9–21.0)	1,204	13.9 (12.9–14.8)	1,379	9.4 (8.7–10.0)	<.001
Non-Hispanic white	20,277	67.2 (66.3–68.1)	7,272	59.8 (58.5–61.0)	4,711	70.1 (68.9–71.4)	8,294	75.0 (73.9–76.0)	
Non-Hispanic black	5,101	11.8 (11.2–12.3)	2,043	12.6 (11.9–13.3)	1,060	10.8 (10.0–11.7)	1,998	11.3 (10.5–12.1)	
Asian	2,078	5.2 (4.9–5.5)	1,164	6.9 (6.4–7.5)	386	4.5 (4.0–5.1)	528	3.4 (3.0–3.9)	
Other race	363	0.8 (0.6–1.0)	156	0.8 (0.6–1.0)	66	0.6 (0.4–0.9)	141	0.9 (0.7–1.2)	
**Age group, y**									
18–24	3,329	12.9 (12.3–13.5)	2,549	22.7 (21.6–23.8)	590	10.1 (9.0–11.1)	190	2.1 (1.6–2.5)	<.001
25–34	5,955	17.6 (17.0–18.2)	4,077	27.7 (26.8–28.7)	1,269	16.4 (15.3–17.5)	609	5.2 (4.7–5.7)	
35–44	5,611	16.9 (16.4–17.5)	3,081	21.5 (20.6–22.4)	1,434	19.3 (18.1–20.5)	1,096	9.4 (8.7–10.1)	
45–54	5,760	18.6 (18.0–19.1)	2,105	15.8 (15.0–16.6)	1,531	22.5 (21.3–23.7)	2,124	19.6 (18.7–20.5)	
55–64	5,728	16.3 (15.8–16.8)	1,213	7.8 (7.3–8.3)	1,304	17.4 (16.3–18.6)	3,211	26.5 (25.3–27.6)	
≥65	7,174	17.8 (17.2–18.3)	765	4.4 (4.0–4.8)	1,299	14.3 (13.3–15.4)	5,110	37.3 (36.2–38.4)	
**Sex**									
Male	14,858	48.2 (47.4–48.9)	6,502	50.6 (49.5–51.6)	3,234	47.6 (46.0–49.1)	5,122	45.5 (44.4–46.6)	<.001
Female	18,699	51.8 (51.1–52.6)	7,288	49.4 (48.4–50.5)	4,193	52.4 (50.9–54.0)	7,218	54.5 (53.4–55.6)	
**Marital status**									
Divorced or separated	6,305	13.5 (13.0–13.9)	1,958	10.1 (9.6–10.7)	1,399	13.4 (12.6–14.3)	2,948	17.8 (17.1–18.6)	<.001
Married	14,583	53.2 (52.4–53.9)	5,879	48.5 (47.4–49.6)	3,406	56.5 (55.0–58.1)	5,298	57.0 (55.9–58.2)	
Single	9,333	27.1 (26.4–27.8)	5,526	39.7 (38.6–40.9)	2,009	24.9 (23.6–26.1)	1,798	12.3 (11.6–13.0)	
Widowed	3,257	6.2 (5.9–6.5)	389	1.7 (1.5–1.9)	592	5.2 (4.6–5.7)	2,276	12.8 (12.1–13.5)	
**Region**									
Northeast	5,617	18.2 (17.5–18.9)	2,302	18.9 (17.8–19.9)	1,292	18.9 (17.5–20.2)	2023	16.9 (16.0–17.8)	<.001
Midwest	6,967	22.7 (21.9–23.5)	2,783	22.0 (21.0–23.1)	1,615	23.5 (22.1–24.9)	2,569	23.1 (21.9–24.4)	
South	12,184	36.4 (35.5–37.3)	4,848	35.2 (34.0–36.4)	2,566	35.0 (33.5–36.5)	4,770	38.9 (37.5–40.2)	
West	8,789	22.7 (21.8–23.5)	3,857	23.9 (22.8–25.0)	1,954	22.7 (21.3–24.0)	2,978	21.1 (20.0–22.2)	
**Employment status**									
Employed	21,412	66.5 (65.7–67.3)	10,817	78.1 (77.2–79.1)	5,235	72.4 (71.0–73.9)	5,360	47.5 (46.3–48.7)	<.001
Unemployed	12,121	33.5 (32.7–34.3)	2,961	21.9 (20.9–22.8)	2,188	27.6 (26.1–29.0)	6,972	52.5 (51.3–53.7)	
**Imputed family income, $**									
0–34,999	13,936	33.5 (32.6–34.4)	5,376	31.7 (30.5–33.0)	2,747	28.9 (27.4–30.3)	5,813	38.7 (37.5–40.0)	<.001
35,000–74,999	9,673	31.9 (31.2–32.7)	4,028	31.6 (30.5–32.6)	2,193	31.8 (30.3–33.2)	3,452	32.5 (31.3–33.7)	
75,000–99,999	3,186	12.3 (11.8–12.9)	1,396	12.7 (11.9–13.5)	770	13.6 (12.5–14.7)	1,020	11.0 (10.2–11.9)	
≥100,000	4,963	22.3 (21.4–23.1)	2,252	24.0 (22.8–25.2)	1,330	25.8 (24.2–27.3)	1,381	17.7 (16.5–18.9)	
**Education**									
≤High school diploma	14,012	40.2 (39.4–41.0)	5,285	37.6 (36.5–38.7)	2,876	37.6 (36.2–39.0)	5,851	45.3 (44.0–46.6)	<.001
Some college or associate degree	10,290	31.3 (30.5–32.1)	4,288	31.7 (30.5–32.8)	2,320	31.4 (30.1–32.7)	3,682	30.8 (29.6–31.9)	
Bachelor’s degree	5,838	18.5 (17.9–19.0)	2,776	21.0 (20.1–21.9)	1,401	19.5 (18.4–20.6)	1,661	14.5 (13.6–15.3)	
Graduate or professional degree	3,282	10.0 (9.5–10.5)	1,375	9.7 (9.0–10.4)	811	11.5 (10.6–12.4)	1,096	9.5 (8.7–10.2)	
**Body mass index, kg/m^2^**									
<25.0	11,852	36.6 (35.9–37.3)	6,139	46.2 (45.0–47.4)	2,597	36.1 (34.7–37.5)	3,116	24.5 (23.6–25.4)	<.001
25.0–29.9	11,268	34.9 (34.2–35.6)	4,473	33.3 (32.2–34.3)	2,578	35.4 (34.1–36.8)	4,217	36.5 (35.5–37.6)	
≥30.0	9,434	28.5 (27.9–29.2)	2,740	20.5 (19.6–21.4)	2,046	28.5 (27.1–29.9)	4,648	39.0 (37.9–40.1)	
**Perceived health status**									
Fair or poor	5,082	12.9 (12.4–13.3)	565	3.5 (3.1–3.8)	719	8.3 (7.6–9.0)	3,798	28.1 (27.0–29.2)	<.001
Excellent to good	28,461	87.1 (86.7–87.6)	1,3221	96.5 (96.2–96.9)	6,705	91.7 (91.0–92.4)	8,535	71.9 (70.8–73.0)	
**Frequent stress and anxiety**									
No	22,690	68.3 (67.6–69.0)	10,838	78.6 (77.6–79.6)	4,863	64.9 (63.4–66.3)	6,989	57.2 (56.0–58.5)	<.001
Yes	10,846	31.7 (31.0–32.4)	2,941	21.4 (20.4–22.4)	2,562	35.1 (33.7–36.6)	5,343	42.8 (41.5–44.0)	
**Fatigue**									
No	28,165	84.6 (84.1–85.1)	12,986	94.1 (93.7–94.6)	6,377	85.6 (84.5–86.6)	8,802	71.7 (70.6–72.7)	<.001
Yes	5,374	15.4 (14.9–15.9)	798	5.9 (5.4–6.3)	1,049	14.4 (13.4–15.5)	3,527	28.3 (27.3–29.4)	

Abbreviations: CI, confidence interval; NA, not applicable.

a Each n reported in the table is not weighted, but all percentages are weighted.

b Rao-Scott χ^2^ tests were used to assess associations between population characteristics and number of chronic conditions.

CAM use was common in the US population. The 4 most frequently used therapies in the past year were multivitamins (52.7%), vitamins (34.8%), minerals (28.4%), and nonvitamin or herbal therapies (17.9%) (Table 2). Adults with multiple chronic conditions reported using on average 2.0 CAM therapies in the last year. Compared with adults with 1 condition or no chronic conditions, adults with multiple chronic conditions reported higher frequency of multivitamin or multimineral use (57.1%), vitamins (42.8%), minerals (37.5%), nonvitamin or herbal therapies (22.0%), chiropractic or osteopathic manipulation (10.1%), massage (9.7%), special diets (3.6%), and acupuncture (1.9%). Conversely, adults with multiple chronic conditions reported using mind–body and movement therapy less often than those with no chronic conditions or one chronic condition (Table 2).

**Table 2:** Use of CAM in the Past 12 Months by Number of Chronic Conditions, National Health Interview Survey, 2012

CAM Index, Mean (SE)	Total Study Population (n = 33,557)		0 Chronic Conditions (n = 13,790)		1 Chronic Condition (n = 7,427)		≥2 Chronic Conditions (n = 12,340)		*P* b
	Mean^a^	95% CI (SE)	Mean^a^	95% CI (SE)	Mean^a^	95% CI (SE)	Mean^a^	95% CI (SE)	
All CAM	1.8	1.8–1.8 (0.02)	1.5	1.5–1.6 (0.02)	1.9	1.9–2.0 (0.03)	2.0	2.0–2.1 (0.02)	<.001
Excluding multivitamins	1.3	1.2–1.3 (0.01)	1.0	1.0–1.1 (0.02)	1.4	1.3–1.4 (0.02)	1.5	1.4–1.5 (0.02)	<.001
Excluding multivitamins, vitamins and minerals	0.6	0.6–0.7 (0.01)	0.6	0.5–0.6 (0.01)	0.7	0.7–0.7 (0.02)	0.7	0.6–0.7 (0.02)	<.001
**Specific CAM therapies**	**N**	**% (CI)^a^**	**n**	**% (CI)^a^**	**n**	**% (CI)^a^**	**n**	**% (CI)^a^**	***P* b**
Multivitamin or multimineral	17,493	52.7 (52.0–53.5)	6,628	48.4 (47.2–49.6)	3,985	54.6 (53.2–56.0)	6,880	57.1 (55.9–58.2)	<.001
Vitamin	11,662	34.8 (34.0–35.6)	3,751	27.6 (26.6–28.6)	2,670	36.8 (35.3–38.3)	5,241	42.8 (41.5 –44.1)	<.001
Mineral	9,891	28.4 (27.7–29.2)	2,979	21.0 (20.1–21.9)	2,225	29.3 (27.9–30.8)	4,687	37.5 (36.3– 38.7)	<.001
Nonvitamin or herbal therapies	5,974	17.9 (17.2–18.6)	1,925	13.6 (12.8–14.4)	1,431	20.0 (18.8–21.3)	2,618	22.0 (20.8–23.1	<.001
Mind–body therapy	4,127	12.5 (11.9–13.0)	1,771	12.8 (12.1–13.6)	1,006	13.9 (12.9–15.0)	1,350	11.0 (10.3 –11.7)	<.001
Chiropractic or osteopathic manipulation	2,993	9.1 (8.7–9.5)	991	7.5 (6.9–8.1)	776	10.7 (9.8–11.6)	1,226	10.1 (9.4–10.8)	<.001
Massage	2,951	8.8 (8.4–9.2)	1,094	7.8 (7.2–8.4)	716	9.4 (8.6–10.3)	1,141	9.7 (9.0–10.4)	<.001
Movement therapy	2,162	6.6 (6.2–7.0)	974	7.2 (6.5–7.8)	584	7.8 (7.1–8.5)	604	5.0 (4.5–5.6)	<.001
Special diets	1,027	3.0 (2.8–3.3)	341	2.4 (2.0–2.7)	266	3.4 (2.9–4.0)	420	3.6 (3.1–4.1)	<.001
Homeopathy	718	2.2 (2.0–2.4)	270	2.1 (1.8–2.4)	185	2.6 (2.1–3.1)	263	2.1 (1.8–2.5)	.1
Acupuncture	604	1.6 (1.4–1.8)	196	1.3 (1.1–1.5)	142	1.8 (1.4–2.1)	266	1.9 (1.6–2.2)	.002
Naturopathy	276	0.7 (0.6–0.8)	96	0.6 (0.5–0.8)	75	0.7 (0.5–1.0)	105	0.9 (0.7–1.0)	.15
Traditional healing	170	0.4 (0.4–0.5)	67	0.5 (0.3–0.6)	35	0.4 (0.2–0.5)	68	0.5 (0.3–0.6)	.70
Craniosacral therapy	109	0.3 (0.2–0.4)	37	0.3 (0.2–0.4)	25	0.2 (0.1–0.3)	47	0.3 (0.2–0.5)	.50
Ayurvedic medicine	96	0.3 (0.2–0.3)	45	0.3 (0.2–0.5)	25	0.2 (0.1–0.3)	26	0.2 (0.1–0.3)	.10

Abbreviations: CAM, complementary and alternative medicine; CI, confidence interval; SE, standard error.

a Each n reported in the table is not weighted, but all averages and percentages are weighted.

b *P* values for association between CAM therapies and number of chronic conditions. Rai-Scott χ^2^ tests and univariable linear regression models used for categorical and continuous variables, respectively.

After controlling for a priori confounders of age, sex, race, family income, employment status, and education, adults with 2 or more chronic conditions were more likely than adults with no chronic conditions to report using multivitamins/minerals, minerals, vitamins, nonvitamin or herbal therapies, mind–body therapies, chiropractic or osteopathic manipulation, massage, and special diets (Table 3). In models adjusted for additional confounding factors, the relationships persisted in all outcome models, and the positive association between multiple chronic conditions and use of movement therapy, acupuncture, and naturopathy became significant (Table 3).

**Table 3:** Association Between CAM Use and Number of Chronic Conditions, National Health Interview Survey, 2012

CAM Modality Outcome Measures^a^								
Unadjusted Models			Minimally Adjusted^b^ Models			Final Adjusted^c^ Models		
No. of Chronic Conditions			No. of Chronic Conditions			No. of Chronic Conditions		
0	1	≥2	0	1	≥2	0	1	≥2

n = 13,790	n = 7,427	n = 12,340	n = 13,790	n = 7,427	n = 12,340	n = 13,790	n = 7,427	N = 12,340
**CAM indexes, β coefficient (95% CI)^d^**								
All CAM								
Ref	0.4^e^	0.5^e^	Ref	0.3^e^	0.6^e^	Ref	0.3^e^	0.6^e^
	(0.3–0.4)	(0.5–0.6)		(0.3–0.4)	(0.5–0.6)		(0.3–0.4)	(0.6–0.7)
Excluding multivitamins/minerals								
Ref	0.3^e^	0.4^e^	Ref	0.3^e^	0.5^e^	Ref	0.3^e^	0.5^e^
	(0.3–0.4)	(0.4–0.5)		(0.2–0.3)	(0.4–0.5)		(0.2–0.3)	(0.5–0.6)
Excluding multivitamins, vitamins, and minerals								
Ref	0.1^e^	0.1^e^	Ref	0.1^e^	0.2^e^	Ref	0.1^e^	0.2^e^
	(0.1–0.2)	(0.1–0.1)		(0.1–0.2)	(0.2–0.2)		(0.1–0.2)	(0.2–0.3)
**Specific CAM therapies, RR (95% CI)^f^**								
Multivitamin or multimineral								
Ref	1.1^e^	1.2^e^	Ref	1.1^g^	1.1^e^	Ref	1.1^e^	1.2^e^
	(1.1–1.2)	(1.1–1.2)		(1.0–1.1)	(1.1–1.2)		(1.0–1.1)	(1.1–1.2)
Mineral								
Ref	1.4^e^	1.8^e^	Ref	1.2^e^	1.4^e^	Ref	1.2^e^	1.4^e^
	(1.3–1.5)	(1.7–1.9)		(1.1–1.3)	(1.3–1.5)		(1.1–1.3)	(1.4–1.5)
Vitamin								
Ref	1.3^e^	1.6^e^	Ref	1.2^e^	1.4^e^	Ref	1.2^e^	1.4^e^
	(1.3–1.4)	(1.5–1.6)		(1.2–1.3)	(1.3–1.5)		(1.2–1.3)	(1.3–1.5)
Nonvitamin or herbal therapies								
Ref	1.5^e^	1.6^e^	Ref	1.4^e^	1.7^e^	Ref	1.4^e^	1.7^e^
	(1.4–1.6)	(1.5–1.7)		(1.3–1.5)	(1.5–1.8)		(1.3–1.6)	(1.6–1.9)
Mind–body therapy								
Ref	1.1^h^	0.9 e	Ref	1.2^e^	1.4^e^	Ref	1.3^e^	1.6^e^
	(1.0–1.2)	(0.8–0.9)		(1.1–1.4)	(1.3–1.6)		(1.2–1.4)	(1.4–1.7)
Chiropractic or osteopathic manipulation								
Ref	1.4^e^	1.3^e^	Ref	1.3^e^	1.4^e^	Ref	1.3^e^	1.4^e^
	(1.3–1.6)	(1.2–1.5)		(1.2–1.5)	(1.2–1.6)		(1.2–1.5)	(1.2–1.6)
Massage								
Ref	1.2^g^	1.2^e^	Ref	1.3^e^	1.8^e^	Ref	1.3^e^	1.9^e^
	(1.1–1.4)	(1.1–1.4)		(1.1–1.4)	(1.6–2.1)		(1.2–1.5)	(1.7–2.2)
Movement therapy								
Ref	1.1^h^	0.7^e^	Ref	1.2^h^	1.2^h^	Ref	1.3^e^	1.3^e^
	(1.0–1.2)	(0.6–0.8)		(1.1–1.4)	(1.0–1.4)		(1.1–1.4)	(1.1–1.5)
Special diets								
Ref	1.5^e^	1.5^e^	Ref	1.5^e^	1.9^e^	Ref	1.5^e^	1.8^e^
	(1.2–1.8)	(1.3–1.8)		(1.2–1.9)	(1.5–2.4)		(1.2–1.9)	(1.5–2.4)
Homeopathy								
Ref	1.2^h^	1.0 h	Ref	1.3^h^	1.3^h^	Ref	1.3^h^	1.4^h^
	(1.0–1.6)	(0.8–1.3)		(1.0–1.6)	(1.0–1.7)		(1.0–1.7)	(1.0–1.8)
Acupuncture								
Ref	1.4^h^	1.5^e^	Ref	1.3^h^	1.6^h^	Ref	1.3^h^	1.8^e^
	(1.0–1.8)	(1.2–1.9)		(1.0–1.8)	(1.2–2.1)		(1.0–1.8)	(1.3–2.4)
Naturopathy								
Ref	1.2^h^	1.4^h^	Ref	1.3^h^	2.0^h^	Ref	1.4^h^	2.4^e^
	(0.9–1.8)	(1.0–2.0)		(0.9–2.0)	(1.3–3.2)		(0.9–2.1)	(1.5– 3.8)
Traditional healers								
Ref	0.8^h^	1.0^h^	Ref	1.1^h^	1.9^h^	Ref	1.1^h^	1.9^h^
	(0.5–1.3)	(0.6–1.5)		(0.6–1.9)	(1.1–3.2)		(0.6–2.0)	(1.1–3.2)
Craniosacral								
Ref	0.8^h^	1.2^h^	Ref	0.7^h^	1.2^h^	Ref	0.7^h^	1.4^h^
	(0.4–1.5)	(0.7–2.0)		(0.3–1.4)	(0.6–2.7)		(0.4–1.5)	(0.6–3.1)
Ayurvedic medicine								
Ref	0.7^h^	0.5^h^	Ref	0.7^h^	0.6^h^	Ref	0.7^h^	0.6^h^
	(0.4– 1.2)	(0.3– 1.0)		(0.4– 1.2)	(0.3– 1.3)		(0.4– 1.3)	(0.3–1.3)

Abbreviations: CAM, complementary and alternative medicine; CI, confidence interval; RR, relative risk.

a Each modality was run as a separate relative risk regression model.

b Adjusted for age, sex, race, income, employment status, and education.

c Adjusted for age, sex, race, income, employment status, education, body mass index, and marital status.

d Values are β (95% CI).

e *P* value <.001 (α = .003 after Bonferroni adjustment for multiple comparisons).

f Values are RR (95% CI).

g *P* value <.003 (α = .003 after Bonferroni adjustment for multiple comparisons).

h *P* value >.003 (α = .003 after Bonferroni adjustment for multiple comparisons).

For the CAM index, after adjustment, adults with multiple conditions used significantly more CAM therapies than adults with no chronic conditions. No collinearity between predictors was observed for the final adjusted models (data not shown). Sensitivity analyses, examining more conservative definitions of CAM, resulted in smaller β coefficients. However, these definitions showed the same overall relationship as the all-inclusive CAM index (Table 3).

## Discussion

Results from the 2012 National Health Interview Survey showed more than half of US adults had at least one chronic condition and over a third had 2 or more chronic conditions. Dietary supplements were used most commonly. In multivariable models we observed that adults with multiple conditions were more likely to report using multiple forms of CAM therapies within the previous 12 months.

Previous studies using the NHIS CAM questionnaire in 2002 and 2007 reported similar associations between specific chronic conditions and CAM use, but no prior analysis has examined use in 13 simultaneous chronic conditions (6,7,10,11,27). In 2002, adults with asthma (7), cancer (6), diabetes (11), and at least one of 5 specific chronic conditions (arthritis, cancer, cardiovascular disease, diabetes, and lung disease) (27) reported higher CAM use than people with no chronic conditions. A study examining both the 2002 and 2007 NHIS data set also found that among adults with diabetes, participants with functional limitations or 3 or more conditions were more likely to use CAM (10). These studies are similar to the results of our analyses, where adults with multiple conditions reported a higher likelihood of CAM use.

Our study differs from previously reported NHIS studies in the definition of chronic condition, the definition of CAM, and in the choice of statistical model. First, prior studies vary in their definitions of chronic conditions. Many of these studies examined singular chronic conditions or a limited number of chronic conditions. Our study has an inclusive definition of multiple chronic conditions. Second, our study examines specific CAM therapies as opposed to other studies, which have focused on combined variables for any CAM therapy. Last, previous studies used odds ratios as compared with risk ratios. The epidemiological convention for point estimates states that when prevalence is more than 10%, the odds ratio will show an overestimated measure of association in comparison to the risk ratio, so the risk ratio should be used (28). Risk ratios were used in this analysis because many of the specific CAM therapies were reported as being used by more than 10% of the population. Although one cannot compare the specific estimates, the general direction of the association is consistent between our analysis and previous analyses. Additionally, these studies report data from the 2002 and 2007 surveys, suggesting this study’s results support an ongoing trend of CAM use in association with chronic conditions. To our knowledge, this is the first study to examine the likelihood of specific CAM modality use by multiple chronic conditions as defined by DHHS (4).

A major strength of this study is the examination of CAM use as separate therapies. CAM is a group of separate behaviors that have differing intensity, effectiveness, and adverse effects. When these behaviors are combined into one overall CAM construct, information is lost regarding the direction of effect for specific therapies. Examining specific CAM therapies allowed us to parse out specific self-care and disease management behaviors among adults with chronic conditions. Our results showed that not all CAM therapies are associated with chronic conditions or multiple chronic conditions and further support the decision to examine CAM use as specific therapies rather than one overarching construct.

There are also limitations to note. First, the NHIS is a cross-sectional study, so temporality between chronic conditions and specific CAM use cannot be determined. One possibility is that chronic conditions influence people to use specific CAM therapies. Conversely, it is also possible that the CAM use precedes the development of a chronic condition. Second, there may be selection bias; the NHIS process excludes hospitalized and institutionalized people, causing an underestimation of adults with chronic illnesses as well as the most severe chronic conditions. This selection bias causes the sample to have proportionally more healthy people than are in the US population and more participants that have the ability to access CAM therapies, causing a bias toward the null. Third, both chronic disease status and CAM use is self-reported, resulting in potential misclassification and recall bias. Lastly, this data set does not include frequency of CAM modality use. If an individual used a modality once in the past year, they would be considered users, as would someone who uses these therapies weekly or daily. This causes an issue of determining what constitutes CAM behaviors. If the users who do not use a modality frequently in truth should be considered nonusers then there is nondifferential misclassification of outcome, biasing the association toward the null. In addition, there may be people with chronic conditions who discontinue CAM within the past year because of factors related to their disease status, which would overestimate the number of regular CAM users.

Multiple chronic conditions increase health care costs not only for the individual but also for the health care system. People with many conditions must navigate the health-care system to coordinate disease management, which often requires regular visits to different medical specialists. This increases the cost for the patient and health-care spending. People with multiple conditions account for approximately 66% of total health care costs (3). DHHS has created 4 goals to improve factors related to multiple chronic conditions, including changes to the health care system; empowering people with multiple conditions by creating community wellness programs; providing clinicians with education, training, and clinical practice guidelines; and improving research practices to include a focus on comorbid conditions as opposed to specific diseases (29).

Research on CAM use among people with comorbid conditions can provide information in support of the DHHS goals. The high use of dietary supplements among people with comorbid conditions is of major importance in disease management because of potential drug interactions. More research is needed to understand the efficacy of supplements and how they interact with standard chronic condition treatments. In addition, we observed high use of practitioner-based CAM. To improve disease management, an open dialogue between CAM practitioners and medical professionals could help improve decisions on care for patients with multiple chronic conditions. Additional research will help provide clinicians with evidence-based guidelines and lower health-care reimbursements. Therapies with an evidence base for improved health outcomes in this population could also be integrated into community wellness programs.

In summary, using data from a population-based sample of US adults, we found that adults with multiple chronic conditions have an increased likelihood of using specific types of CAM, including dietary supplements, mind–body therapies, chiropractic or osteopathic manipulation, massage, movement therapies, special diets, acupuncture, and naturopathy. Because adults with chronic conditions have an increased likelihood of using specific CAM therapies, in the face of unclear evidence, it is important to conduct CAM research on people with multiple chronic conditions and not only populations with specific diseases. Chronic condition management is an integral part in improving mortality and reducing illness among people with chronic conditions. Further research should focus on the efficacy of these therapies in individuals with multiple chronic conditions and on interactions with standard chronic disease treatments.
